# The Impact of a University Counselling and Psychological Support Service Focused on Positive Resources and Student Well-Being

**DOI:** 10.3390/bs16030410

**Published:** 2026-03-11

**Authors:** Lucrezia Perrella, Patrizia Patrizi, Gian Luigi Lepri, Maria Luisa Scarpa, Ernesto Lodi

**Affiliations:** Department of Humanities and Social Sciences, University of Sassari, 07100 Sassari, Italy; patrizi@uniss.it (P.P.); gianluigi.lepri@me.com (G.L.L.); mlscarpa@icloud.com (M.L.S.); elodi@uniss.it (E.L.)

**Keywords:** university counselling, university students, well-being, psychosocial positive resources, academic satisfaction

## Abstract

Today, university counselling services play a crucial role in creating places where personal and professional skills can be developed. Universities provide an environment where people can grow as individuals and improve their quality of life. The aim of the study was to evaluate the impact of a counselling service that uses positive psychology as a theoretical and practical framework on students’ well-being and positive resources. Methods: Seventy students aged between 19 and 54 (M = 24.2; SD = 5.87), of whom 68.6% were women and 31.4% were men, participated in 10 psychological counselling sessions. The sessions focused on academic and general well-being, non-intellectual skills related to academic performance and satisfaction (e.g., academic self-efficacy, motivation, reaction to failure, time management), as well as positive resources (e.g., hope, resilience, courage). Participants completed a questionnaire protocol on these variables before and 6 months after the intervention. Results: The results show a significant increase in almost all indices of general and domain-specific well-being and in positive psychosocial resources. The participants themselves stated that the counselling intervention produced significant changes in their lives in general and as university students. Conclusions: The results seem to suggest that structuring counselling programmes with a positive, well-being-oriented perspective can promote students’ professional and personal development. Building psychological support environments can guide everyone on the path to maximising their potential in life and professional trajectories. The university services must pay constant attention not only to student performance but, above all, to improving their quality of life, preventing distress and promoting well-being.

## 1. Introduction

Since the late 1990s, counselling services have become increasingly important in universities (Francis & Horn, 2017), and today we are seeing a growing demand for interventions to support the mental health and well-being of university students (Duffy et al., 2023; Sharma et al., 2023; Zammitti et al., 2023).

There are several studies in the literature that address the importance of counselling services, particularly about supporting students in building career paths (S. D. Brown & Lent, 2008) and life trajectories (Savickas, 2013). The purpose of these services is not only to support students in identifying aspects of university life that may represent obstacles to their career development. The aim is also to support them in improving the individual and contextual resources that promote high-quality-of-life in the environments that people value (Duffy et al., 2023). The mission of psychological counselling consists specifically in identifying practices that help people increase their well-being and live their lives by optimising their resources and functioning, both individually and socially (Corey, 2016; Katajavuori et al., 2023). Consequently, university counselling services should intervene through counselling and psychological support to promote and develop student well-being. The aim should be to accompany them on a path of personal and social growth, promoting recognition of the meaning of their experiences and life lessons in their studies and everyday life (Sharma et al., 2023). These needs have become even more relevant to cope with the many changes related to the COVID-19 pandemic. The latter has amplified experiences of precariousness and instability, putting a strain on resilience, restructuring and coexistence with emotions and moods that have generated various forms of psychological distress. These include depression (Haliwa et al., 2021; Halliburton et al., 2023; Liu et al., 2023), anxiety (Mahamid et al., 2025; Russell et al., 2024), stress (Haliwa et al., 2021; Chachula & Ahmad, 2022; Voltmer et al., 2021; von Keyserlingk et al., 2022), perceived loneliness (Cipolletta et al., 2025; Mahamid et al., 2025), and uncertainty about the future (Cipolletta et al., 2025; Russell et al., 2024). These aspects represent real obstacles to personal and professional fulfilment.

From the perspective of positive psychology, well-being indices (i.e., life satisfaction, flourishing, and academic satisfaction) and psychosocial resources, e.g., motivation, resilience, self-efficacy, hope, and optimism, play a fundamental role in the prevention and promotion of health and well-being (Seligman, 2002; Seligman & Csikszentmihalyi, 2000). Despite growing attention to the psychological well-being of university students, there are still few studies that systematically assess the impact of university counselling services on students’ overall well-being and on indicators of subjective and academic well-being (Pidgeon et al., 2014; Kern et al., 2014; Turner et al., 2017). Furthermore, most studies focus on clinical interventions rather than interventions inspired by positive psychology, i.e., centred on strengthening personal and relational resources (Oades et al., 2016; Lomas et al., 2020). A further gap is the lack of evidence on the effectiveness of university counselling services in the European context, where these types of interventions are increasingly assuming a strategic role in university policies (Di Fabio & Kenny, 2016; Cipolletta et al., 2025). Consequently, this study aims to help fill these gaps. It aims to evaluate the impact of a university psychological counselling service inspired by the theoretical model and interventions associated with positive psychology. The study will analyse how this intervention can influence indicators of subjective, academic and psychosocial well-being. It will also seek to provide new empirical evidence useful for the design of effective support programmes aimed at promoting well-being.

## 2. Literature Review

In recent years, the university environment has become increasingly central in promoting students’ mental health and well-being (Hernández-Torrano et al., 2020; Worsley et al., 2022). This has also been achieved through the integration of counselling and psychological guidance services (Collins et al., 2025; Elkin et al., 2025). Students who access these services often present forms of distress that manifest themselves through study blocks, exam anxiety, and relationship difficulties (Savarese et al., 2023). These forms of distress have a negatively impact their educational path and personal well-being. Several studies show that counselling interventions, both individual and group, improve life satisfaction (Odacı & Çelik, 2017; Yang et al., 2023), self-esteem (Blau & DiMino, 2018; Mao et al., 2020), perceived social support (Julal, 2015; Stallman et al., 2017), motivation (Karaman et al., 2017) and a sense of effectiveness in study and life (Zammitti et al., 2023). Counselling programmes seem to work not only to promote the ability to regulate emotions and manage academic stress (A. P. Brown et al., 2013; Wu et al., 2021) but also as preventive interventions against the development of psychopathologies (Saijonkari et al., 2023). As demonstrated by Scruggs et al. (2023), psychological support has a direct impact on the academic performance of students experiencing psychological distress. Flourishing, defined as the perception of one’s ability to fully realise one’s potential and qualities (Keyes, 2007; Seligman, 2012), is positively associated with psychological well-being (Lodi et al., 2022) and academic performance (Shdaifat et al., 2024), with effects on reducing anxiety, promoting self-efficacy (Wu et al., 2021) and resilience (Deng et al., 2020). Similarly, the importance of hope in strengthening internal locus of control, understood as the tendency to consider oneself capable of controlling the effects of one’s behaviour (Weiner, 1986), and planning for the future (Karaman et al., 2017) has emerged. The ability to manage emotions is fundamental in academic life (Öztekin et al., 2025), as effective emotional regulation can help reduce anxiety and stress (Keskiner et al., 2025) and improve resilience and interpersonal relationships (Gross, 2002; Huai et al., 2025). Enhancing study methods is also essential: counselling programmes focused on stress management and academic work organisation help students develop effective strategies for coping with university demands (Regehr et al., 2013; Yusoff et al., 2021). Study skills—including planning, concentration and time management—have been found to be strongly correlated with academic success (Credé & Kuncel, 2008; Fu et al., 2025). Furthermore, the quality of relationships, whether with family, peers or teachers, is crucial (Feng & Zhang, 2022; Ruihua et al., 2025), as it increases overall well-being and mental health, resilience, and life satisfaction (Ruihua et al., 2025; Smith, 2011).

## 3. Counselling and Psychological Support Service

### 3.1. Work Methodology

The counselling service is a self-referral service, formally established at the University of Sassari, and is free for the whole student population. Students autonomously access to the service by filling out a booking form on the university website. Students are then contacted by the counsellor to arrange the first appointment and begin counselling. Students attend a maximum of 10 counselling sessions.

Before starting work for the service, each counsellor receives 24 h of theoretical and practical training from qualified experts in positive psychology and professional counselling. The aim is to train counsellors in programmes and techniques to be used during counselling sessions, thereby increasing the variables targeted by the intervention. For example, the training modules covered “how to increase students general well-being and satisfaction in specific areas” (e.g., focus on positive emotions, study methods, time management, academic self-efficacy, reaction to failure); “how to increase students positive psychosocial skills” (e.g., focus on courage, resilience, hope and optimism); and “how to increase students’ positive relationships” (e.g., with other students and professors).

Before the counselling process begins, each student is asked to complete a questionnaire on well-being and the variables that promote it. Six months after the end of the counselling process, a follow-up study is conducted. The aim of the survey is to identify the attitudes and behaviours that students adopt and the feelings they experience during their university career, assessing three general macro-areas: (1) personal well-being and academic satisfaction; (2) “non-intellectual” academic skills associated with university performance and satisfaction; and (3) psychosocial resources linked to theories that originate mainly from positive psychology. By completing the questionnaire, students receive a brief profile (see Supplementary Materials “Student Profile Example”) highlighting their strengths and/or areas for improvement. Student profiles enable counsellors to be as effective as possible in supporting them in their needs, developing precisely those “positive” strengths that will enable them to cope with adversity and promote well-being.

### 3.2. Structure of the Counselling Sessions

The first two meetings are dedicated to listening to the students’ motivations for enrolment and their history. From the third to the eighth meeting, work is done on the positive variables chosen by the counsellor and student from the questionnaire profile. The last two meetings are dedicated to closing the process and reflecting on the results achieved. The counselling programme is personalised based on the student’s profile. The profile is read and analysed by the counsellors and students to build the programme together. The programme involves experimenting with strategies primarily aimed at promoting variables within areas of possible improvement. For example, optimism-based approaches are used to promote personal well-being. Students are encouraged to identify negative thoughts that arise in times of difficulty and transform them into positive beliefs through alternative explanations of events known as “reatributions” (Shatté et al., 1999). Work on courage is done by presenting stories of courage to stimulate and strengthen courageous actions, and by reflecting on the benefits of such actions. Examples include stories of: (a) courageous actions in the face of risks (Pury & Saylors, 2017; D. Putman, 1997); (b) courageous actions taken in the face of threats to personal well-being and psychological stability (R. J. Putman, 1996). Other examples include Hope Mapping techniques (Snyder, 1994) to promote hope, or the resilience diary to design alternative scenarios (Savickas et al., 2009).

## 4. Aim of the Study and Hypothesis

The study aimed to verify the impact of counselling programmes on students’ well-being and positive resources. The results report data from a follow-up conducted six months after the end of the counselling programme.

Based on the theoretical framework and previous empirical evidence, the following hypotheses were formulated:

**H1.**  *Participants will report significantly higher levels of courage after completing the counselling programme.*

**H2.**  *Participants will report significantly higher levels of optimism and hope after completing the counselling programme.*

**H3.**  *Participants will report significantly higher levels of future orientation and resilience after completing the counselling programme.*

**H4.**  *Participants will report significantly higher levels in all sub-dimensions of academic satisfaction after completing the counselling programme.*

**H5.**  *Participants will report significantly higher levels in all areas of the college competencies scale after completing the counselling programme.*

**H6.**  *Participants will report significantly higher levels of life satisfaction after completing the counselling programme.*

**H7.**  *Participants will report significantly higher levels of flourishing after completing the counselling programme.*

## 5. Materials and Method

### 5.1. Participants

Seventy Sardinian students aged between 19 and 54 (M = 24.2; SD = 5.87), of whom 48 (68.6%) were women, and 22 (31.4%) were men. Of these 70, 35 (50%) were enrolled in a Bachelor’s degree programme, 6 (8.6%) in a Master’s degree programme, 25 (35.7%) in a Single-cycle Master’s degree programme and 4 (5.7%) in a Doctorate/Master’s/Specialisation programme. The students mainly come from Law (22.9%), followed by Humanities and Social Sciences (17.1%), Chemical Sciences (11.4%), Biomedical Sciences (10%), Economics and History (8.6%), Veterinary Medicine (7.1%), Medicine, Surgery and Pharmacy (5.7%), and Agriculture and Architecture (4.3%). A total of 98.6% of participants study full-time. A total of 61.4% are non-resident students, while 38.6% are resident students. The participants are mainly Italian nationals (92.9%). Some 18.6% work while studying. Table 1 summarises these data.

### 5.2. Instruments

Socio-demographic section: age, gender, course attended, course year, department of affiliation, student status, two single items about the importance of the university (1 = not important at all; 5 = extremely important) and satisfaction with academic performance (1 = not satisfied at all; 5 = extremely satisfied).

Courage Measure (Norton & Weiss, 2009): The Italian version of the Courage Measure (Ginevra et al., 2020), adapted from the short version by Howard and Alipour (2014), consists of 6 items structured on a 7-point Likert response scale (1 = never; 7 = always). The measure demonstrated good level of internal consistency (Cronbach’s alpha = 0.88).

Vision of the future (Ginevra et al., 2017): Assesses, with 22 items on a 5-point Likert scale (1 = it does not describe me at all; 5 = it describes me very well), dimensions such as optimism (Cronbach’s alpha: 0.84), negative vision of the future (Cronbach’s alpha: 0.81), and hope (Cronbach’s alpha: 0.84). In this study, only the 2 sub-dimensions measuring optimism and hope were used.

Designing my future (Santilli et al., 2015): Assesses, with 22 items on a 5-point Likert scale (1 = it does not describe me at all; 5 = it describes me very well), dimensions such as future orientation (Cronbach’s alpha =0.88) and resilience (Cronbach’s alpha = 0.83).

College Satisfaction Scale (C-Sat; Lodi et al., 2017): Measures students’ satisfaction with the university through 20 items on a five-point Likert scale (from 1 = not at all; 5 = completely). The scale measures five dimensions of academic satisfaction: the choice of course of study, services, relationships, study methods, and usefulness for career path. In the first validation study (Lodi et al., 2017), all C-Sat scales showed good reliability (with omega indices between 0.80 and 0.92). 

College Competencies Scale (C-Comp; Boerchi et al., 2021): Contains 48 items on a five-point Likert scale (1 = not at all; 5 = entirely). It is designed to assess 12 non-cognitive factors involved in academic performance and satisfaction, grouped into three macro-areas: (1) study area: intrinsic motivation, extrinsic motivation, time management, dedication to study; (2) self-area: learning assessment, academic self-esteem, academic self-efficacy, reaction to failure, emotional control; (3) relationships area: family relationships, student relationships, relationships with professors. The Cronbach’s alpha of the validation study is 0.80.

Satisfaction with Life Scale (SWLS; Diener et al., 1985): In the validated Italian version (Di Fabio & Gori, 2016), assesses overall life satisfaction through 5 items on a 7-point Likert scale (1 = strongly disagree; 7 = strongly agree). The Cronbach’s alpha of the validation study is 0.88. 

The Flourishing Scale (Diener et al., 2010): In the Italian version validated by Di Fabio (2016), the scale consists of 8 questions on a seven-point Likert scale (1 = strongly disagree; 7 = strongly agree) and is designed to measure the meaning and purpose of life. The Cronbach’s alpha of the validation study was 0.88.

At the beginning, we asked participants an open-ended question about the reason for participating in the counselling programme. At the end of the protocol, so only for the follow-up administration, we used three additional questions (two qualitative and one quantitative): (1) whether the programme carried out at the Service has led to changes in their university life; (2) whether the programme carried out at the Service has led to changes in their life in general; (3) how effectively the service responded to their motivation for accessing it (10-point Likert scale from 1 = not at all to 10 = completely).

### 5.3. Study Design

This study adopted a quasi-experimental design with pre-intervention measurement and a six-month follow-up. The study did not include an immediate assessment at the end of the counselling process but only a follow-up after six months. For mainly ethical reasons, it was not possible to include a control group.

### 5.4. Procedure

The students gave their consent to participate in the research and, aware that the general results would be published, completed an online survey via a Google form. The questionnaire, completed individually, took approximately 20 min. Participants were free to withdraw from the study at any time. To ensure anonymity, each student was assigned an alphanumeric code. It should also be noted that the students’ responses were shared with the counsellor, with the participants’ consent.

Six months after the end of the programme, a new follow-up questionnaire is completed, which includes the same quantitative tools used in the first questionnaire, as well as three additional questions at the end of the protocol.

The responses to the qualitative questions were transferred to a Microsoft Excel v16.102 (25101223) file for analysis. For the analysis, the first version of the form was created based on 50% of the responses and then verified for the remaining 50% by two independent coders (Losito, 2002). The two independent experts coded the responses according to the categories constructed, achieving a high level of inter-rater agreement (k = 0.92) (Cicchetti & Allison, 1971; Landis & Koch, 1977; McHugh, 2012).

The survey followed the ethical rules of the Italian Psychological Association, the Helsinki Declaration, and the Code of Ethics of the National Order of Italian Psychologists, and the ethical commission of the University of Sassari approved it (approval date 6 December 2022, n◦ project no. 2022-UNSSCLE-0061755).

### 5.5. Data Analysis

The software used for data analysis was IBM SPSS Statistics, version 29.0, used to calculate descriptive statistics and perform paired *t*-tests to assess differences in means between pre-programme and post-programme. An independent-samples *t*-test was conducted on both the pre-test and post-test to examine differences in students’ initial levels of the variables under study based on socio-demographic characteristics, as well as to verify whether students responded differently to the intervention. Given the large number of *t*-tests, multiple-comparison correction procedures were applied to control for Type I error inflation. *p*-values were corrected using both the Benjamini–Hochberg False Discovery Rate procedure (q = 0.05; Benjamini & Hochberg, 1995) and the Holm–Bonferroni procedure (Holm, 1979), which controls for the family Type I error. Normality was assessed through visual inspection of the difference-score histograms (Ghasemi & Zahediasl, 2012; Gosselin, 2024), which did not indicate substantial deviations from normality. Given the sample size and the robustness of *t*-tests on paired samples for moderate deviations from normality, parametric analyses were considered appropriate (Ghasemi & Zahediasl, 2012; Lumley et al., 2002). To assess the potential impact of systematic attrition due to dropout rates, baseline differences between participants who completed follow-up and those who did not were examined. Independent samples *t*-tests were conducted on all variables in the study (N = 26), and chi-square tests were conducted for categorical socio-demographic variables. A repeated-measures ANOVA was conducted to verify significant differences in the variables under study between the pre-test and post-test, and to examine the effects of socio-demographic variables and their interactions with time. To assess whether students from different courses of study could be considered as a single sample, further analyses were conducted on all the variables under study. For each variable, linear regression models were applied to the pre-post difference scores, with the educational pathway (Bachelor’s degree vs. Master’s degree/Doctorate/Master’s/Specialisation) as the predictor variable.

We also calculated Cohen’s d index to assess effect size. To analyse the impact of the effect size, the following ranges were used: 0.20 small, 0.50 medium, 0.80 large (Cohen, 1988). Microsoft Excel version 16.102 (25101223) was used to analyse the responses to the qualitative questions.

## 6. Results

The 70 participants are among those who responded to the follow-up survey out of a total of 123 students who completed the counselling programme. Thus, 53 students did not complete the survey. All 70 students attended 10 psychological counselling sessions. About dropout rates, a statistically significant difference emerged only for flourishing (t(121) = 2.28, *p* = 0.024, d = 0.41), with participants who completed the study reporting slightly higher baseline levels. However, no other significant differences were observed. Given the number of comparisons performed, this isolated finding may reflect random variation rather than systematic attrition bias. Overall, the pattern of results does not suggest substantial selective dropout, although the findings on flourishing should be interpreted with caution.

Initial differences between socio-demographic groups and any differences in responses to the intervention were analysed. Compared to the pre-test, the analysis revealed no statistically significant differences between the two groups on any of the variables, except for time management between working and non-working students (t = −2.47; *p* = 0.016). Compared to the post-test, no differences emerged by gender. The only statistically significant differences emerged between on-campus and off-campus students on the variables of prosperity (t = 2.48; *p* = 0.015) and future orientation (t = 2.12; *p* = 0.037). Neither the pre-test nor the post-test included the nationality variable due to the significant disproportion between the two groups (Italian = 65; foreign = 5). In all variables examined in the linear regression model, the educational path students followed did not significantly affect pre–post change scores, indicating comparable changes related to the intervention across all courses of study. Finally, a repeated-measures ANOVA was conducted to assess differences between the pre-test and post-test and the effects of socio-demographic variables and their interactions with time. The analyses did not reveal any main effects or significant interactions among the variables considered.

As can be seen from Table 2, in response to the question “What is the reason you are requesting counselling?”, most of the answers concerned the need to manage emotions effectively, for example by contrasting anxiety or panic (51.4%), followed by improving psychosocial and academic skills to continue or complete their studies, for example by improving self-esteem or the quality of relationships (24.3%), addressing issues that affect their academic career (10%), improving study methods (5.6%), improving integration and adaptation to a new educational and social environment, and choosing a course of study at the end of current studies (both 4.3%).

After the counselling programme, students showed a statistically significant increase in four of the five sub-dimensions of the academic satisfaction (C-Sat): “Satisfaction with choice” (*p* = 0.002), “Satisfaction with relationships” (*p* = <0.001), “Satisfaction with study” (*p* = <0.001), and “Satisfaction with the usefulness of the course of study” (*p* = 0.021). Only the sub-dimension “Satisfaction with services” did not show statistically significant increases (*p* = 0.062). After applying the Holm–Bonferroni correction for multiple comparisons, the increase observed for “Satisfaction with the usefulness of the course of study” did not remain statistically significant. The effect size analysis highlights that the greatest impact was on the sub-dimension of “Satisfaction with studies”, followed by the sub-dimensions “Satisfaction with relationships” and “Satisfaction with choice”.

Flourishing (*p* = 0.001) and life satisfaction (*p* = <0.001) also showed a statistically significant increase. For both dimensions, the effect size was medium-high, with the greatest impact on life satisfaction.

Statistically significant increases were found in nine of the 12 non-cognitive factors involved in academic performance and satisfaction (C-Comp): students and professors relationships (*p* = <0.001), reaction to failure (*p* = <0.001), learning assessment and time management (*p* = <0.001), dedication to study (*p* = 0.021), academic self-esteem (*p* = <0.001), and academic self-efficacy and emotional control (*p* = <0.001). The dimensions of family relationships (*p* = 0.120), intrinsic motivation (*p* = 0.094) and extrinsic motivation (*p* = 0.131) have not increased in a statistically significant way. After applying the Holm–Bonferroni correction for multiple comparisons, the increase observed for the dimension “dedication to study” was no longer statistically significant. Regarding the effect size, medium-high coefficients were found for academic self-esteem, teacher–student relationships, emotional control, learning assessment, reaction to failure, academic self-efficacy and time management.

Scores relating to positive resources, such as courage, optimism, hope and resilience, showed a statistically significant increase between the initial assessment and the follow-up (*p* = <0.001), while the change in future orientation was not significant (*p* = 0.102).

Satisfaction with performance showed a statistically significant increase between the initial assessment and the follow-up (*p* = <0.001). Instead, the importance of university in their lives did not show significant scores (*p* = 0.106). The effect size value for satisfaction with performance is medium-high.

These results are shown in Table 3, Table 4, Table 5, Table 6 and Table 7.

As can be seen from Table 8, in response to the question “Did the counselling provided by the Service lead to changes in your university life?”, 80% of participants answered “Yes”, and 20% answered “No”. About the “Yes” responses, the changes reported relate to improved academic self-esteem (44.3%), improved study methods (20%), better ability to manage anxiety before exams (17.1%), and career-related decision-making (10%) (e.g., choice of postgraduate course). In response to the question “Has the counselling provided by the Service led to changes in your life in general?”, 77.1% of participants answered “Yes”, and 22.9% answered “No”. Of those who answered “Yes”, 58.6% reported personal growth in terms of self-improvement in various areas of life; 24.3% reported an improvement in their ability to manage anxiety; and 5.7% began psychotherapy.

In response to the question “How effectively did the service respond to your motivation for accessing it?” (Table 9), on a scale of 1 to 10, 17.1% answered “10 = completely”, 28.6% gave a value of 8, 21.4% gave a value of 7, and 8.6% gave a value of 9. The average response is 7.21 (DS = 2.27).

## 7. Discussion

The aim of this study was to assess whether the counselling programme, based on the theoretical and practical model of positive psychology, had a positive impact on the subjective, academic, and psychosocial well-being of the students involved. The study included a pre- and post-intervention assessment six months after the conclusion of the counselling programme. Not including an immediate assessment at the end of the counselling process was a methodological choice. The variables investigated represent core constructs of positive psychology, which therefore tend to change and consolidate in the medium term rather than immediately (Seligman et al., 2005; Lyubomirsky et al., 2011; Parks & Schueller, 2014). Furthermore, an assessment immediately after the intervention may be influenced by transient factors related to the emotional experience of counselling, increasing the risk of overestimating or distorting perceived effectiveness (Kazdin, 2007; Cuijpers et al., 2014). The six-month follow-up, on the other hand, allows for an assessment of the actual maintenance and integration of benefits into students’ daily lives, in line with the goals of promoting sustainable well-being in positive psychology (Sin & Lyubomirsky, 2009; Bolier et al., 2013). For ethical reasons, it was not possible to include a control group. The control group would have been composed of students who had requested access to the counselling service and whom we would have had to deny the opportunity to start the programme. Furthermore, it would have been unethical to offer students alternative treatment using methods other than those characteristics of the counselling service. According to the ethical principles of the APA (2017), professionals and researchers must protect the individuals’ well-being and must not deny potentially beneficial interventions. As noted by Shadish et al. (2002), when random assignment or the creation of a control group is not feasible for ethical or practical reasons, quasi-experimental designs are a methodologically valid alternative. For these reasons, the use of a single-group pre–post design is consistent with a practice-based evidence approach (Barkham & Mellor-Clark, 2003), which values research conducted in real-world psychological service delivery settings while maintaining methodological rigour and practical relevance.

Regarding the dropout rate, as suggested by methodological literature, attrition threatens internal validity mainly when it is associated with systematic baseline differences between participants who remain in the study and those who dropout (Shadish et al., 2002). In the present study, only the flourishing variable reached statistical significance. In the context of multiple statistical tests, isolated significant results may reflect an increase in Type I error rather than true systematic differences (Benjamini & Hochberg, 1995). Consequently, the observed pattern does not indicate substantial selective attrition, although the results related to flourishing should be interpreted with caution.

Given the large number of pairwise comparisons, a conservative analytical strategy was adopted, applying multiple comparison–correction procedures to control for the risk of false positives. Initially, applying the FDR correction confirmed the initial results mode. With the Holm–Bonferroni correction (Holm, 1979), all variables kept statistical significance except for the “utility” sub-dimension of the C-Sat scale and the “dedication to study” dimension of the C-Comp scale, which proved to be more sensitive to conservative control of Type I error. Both correction procedures confirmed the robustness of the main results. Finally, additional analyses did not suggest systematic differences in intervention-related change across educational paths.

Overall, the findings provide strong support for the hypothesised benefits of the intervention. Students who participated in the counselling programme showed significant improvements in the following: (a) almost all dimensions of academic satisfaction, particularly in satisfaction with the choice of course of study, relationships and study methods; (b) courage; (c) optimism and hope; (d) resilience; I life satisfaction; (f) non-cognitive factors involved in academic performance and satisfaction, in particular (1) study area, i.e., time management; (2) self-area: learning assessment, academic self-esteem, academic self-efficacy, reaction to failure, emotional control; and (3) the area of relationships, such as student and professor relationships; (g) satisfaction with performance. In contrast, future orientation did not show a statistically significant change over time. The overall increase in well-being indices, such as life satisfaction, flourishing and academic satisfaction, may be due to the fact that the counselling process provided students with tools to: improve their ability to set and achieve goals (hope); build positive scenarios (optimism); deal with situations with courage; be confident in their ability to organise and take the necessary actions to deal with specific situations and tasks (self-efficacy). Resilience, an important factor in flourishing, may also have played a significant role in this, as it allows people to maintain their well-being even in difficult situations. All of this seems to converge in the result that the greatest impact was on students’ general perception of themselves as students (academic self-esteem). Flourishing showed a significant increase at follow-up. This could suggest that the counselling programme may have contributed to strengthening students’ perceptions of meaning, purpose and personal growth. However, comparisons with baseline values revealed slightly higher initial levels of flourishing among participants who completed the study. Although this difference was isolated and may reflect random variation, this result should be interpreted with caution.

From a qualitative point of view, the students who participated in the programme stated that the counselling provided by the Service led to several changes in both their university life and their life in general. In particular, the most significant positive changes that students recognise in the university environment concern academic self-esteem, study methods and the ability to manage emotions before exams. Personal growth in terms of self-improvement in various areas of life, such as emotional, psychological, professional and relational, seems to be the most significant change that students report in relation to their lives, along with an improvement in their ability to manage anxiety. Integrating quantitative and qualitative data can be an important research strategy for reflecting the complexity of human beings in their life contexts (Creswell & Plano Clark, 2018; Tashakkori & Teddlie, 2010).

These results are in line with current scientific literature, for example with the literature on positive psychology (Seligman & Csikszentmihalyi, 2000; Snyder, 1994), which emphasises how the enhancement of personal resources promotes subjective well-being and the ability to cope with academic challenges. They are also in agreement with the study by Tinella et al. (2025), which suggests that counselling interventions in universities can improve psychological resources such as emotional regulation and self-efficacy, which are fundamental for improving students’ life satisfaction and academic performance. Similarly, the study by Ross et al. (2022) also reported positive results from a counselling programme with university students, particularly about resilience and, consequently, improved academic satisfaction and overall student well-being. Our results are also in line with those of Shan and Xu (2025), who demonstrated that a counselling intervention focused on promoting hope and optimism helped university students manage emotions (such as anxiety and stress) by increasing levels of psychological flourishing. Finally, research by Lee et al. (2025) has shown that counselling programmes that support students’ self-efficacy and goal-setting skills can play a key role in improving their satisfaction with both the academic and personal aspects of their lives.

Regarding areas that did not show improvement, such as intrinsic and extrinsic motivation, this may be because the counselling programme involves 10 sessions per student, which may not be enough to achieve complex changes in motivation. In fact, on a speculative level, one might think that these changes refer to processes of internalisation and stabilisation (Deci & Ryan, 2000) that sometimes clash with a possible resistance to change in modifying deeply rooted beliefs and behaviours and therefore require long-term processes. In addition, the baseline intrinsic and extrinsic motivation scores of the study participants were well below the theoretical upper limit of the scale. Therefore, it is unlikely that the ceiling effect can explain the non-significant results. It is interesting to note that future orientation did not show a statistically significant change, despite improvements in hope and optimism. Although these constructs are theoretically related, they are not overlapping constructs and have different levels of stability and modifiability. Hope and optimism primarily reflect expectation-based components, which may be more sensitive to short-term counselling interventions (Scheier & Carver, 1985; Snyder, 2002). In contrast, future orientation involves broader and more stable patterns of long-term planning and behavioural regulation (Seginer, 2009; Zimbardo & Boyd, 1999). It is therefore possible that a ten-session intervention was sufficient to improve hope and optimism but not to produce measurable changes in more structurally entrenched future-oriented dispositions. These results are also in line with other studies in the literature (e.g., Bozzato, 2024; Issa & Al-Hamdani, 2022; Parrello et al., 2024).

From a professional practice perspective, this study’s results can confirm the importance of integrating counselling and psychological support programmes into university services. These interventions can not only improve subjective well-being but can also enhance the transferable skills that are useful for academic and professional success. Recent studies (e.g., Mahmood et al., 2025) have highlighted the role of educational contexts in shaping professional aspirations and transferable skills relevant to contemporary career paths. Our findings suggest that counselling services can play a complementary role to the university by strengthening psychological resources (e.g., self-efficacy, hope, resilience) that enable students to take full advantage of these educational opportunities and translate skills into sustainable life and career paths. In addition, highly personalised work on strengths and non-intellectual skills related to academic performance and satisfaction, considered fundamental to the individual’s well-being within the therapeutic alliance with the counsellor, may have contributed to the overall increase in well-being levels across all areas.

This study can contribute to the literature on university counselling as it goes beyond the model of reducing distress by examining the enhancement of positive psychological resources and academic skills. Furthermore, by integrating well-being with the dimensions of life and career development, it can offer a broader framework for understanding the role of counselling services in higher education contexts.

## 8. Limitations

One of the main limitations of this study is the absence of a control group. This is due to both practical and, above all, ethical reasons (APA, 2017; Shadish et al., 2002), which did not even allow for the consideration of study designs with waiting lists or stepped-wedge designs. However, to limit the effects of potential distortions and ensure the internal validity of the study, several measures were taken, such as limiting time between the pre-test and post-test and using validated instruments.

The dropout rate represents another potential limitation. The comparison showed a statistically significant difference only about flourishing. However, no other differences emerged between the remaining variables. Given the number of comparisons made, this isolated result may reflect a Type I error rather than a systematic bias due to attrition. Future studies should implement strategies to reduce loss to follow-up.

Furthermore, no evaluation was carried out at the end of the counselling process; instead, a follow-up was conducted six months after the programme ended. Although this was a methodologically sound choice, future research on this topic will need to conduct longitudinal studies. Finally, although this was not the purpose of the study, we do not have data on the specific variables addressed in each of the 70 programmes. The lack of uniformity in the counselling programme stems from the subjective establishment of work objectives and positive psychology practices in the agreement between the counsellor and the student. On the one hand, this limits the generalisability of the results and the replicability of the study. The training model used for consultants could be the same one used in subsequent studies, allowing comparison with our results. At the same time, this can be considered a strength. The programmes are built on each student’s specific concrete needs and are therefore highly personalised.

Self-assessment measures were used, which may have led to social desirability bias and influenced participants’ responses. Furthermore, the sample is small and comes from only one Italian region, which limits the generalisability of the results. Furthermore, the sample is unbalanced with respect to socio-demographic variables such as gender, which may have influenced the results. On the other hand, the gender composition reflects the gender imbalance among students enrolled at the University of Sassari (57.56% women and 42.44% men).

Finally, the results may have been influenced by maturation effects, regression to the mean, or the academic calendar; for example, the longer time spent at university may have improved their level of awareness of resources and well-being.

## 9. Conclusions

Programmes such as the one presented in this study may be important to support students’ professional and personal development. Sensitivity to contexts, not only in terms of student performance but above all in terms of their quality of life, can grow and transform into concrete improvements. The aim is to ensure inclusive contexts, prevent discomfort, and promote well-being and individual, collective, and contextual empowerment. Creating contexts that are sensitive to well-being (and that generate well-being) can mean creating the best possible “environmental” conditions for everyone to express their full potential in developing their careers. Positive psychology, as the results of this study seem to have highlighted, can offer an appropriate theoretical and practical framework to make this possible. The results highlight the potential role of university counselling services based on positive psychology, as they seem to lead to improvements in various personal and academic resources of students.

## Figures and Tables

**Table 1:** Socio-demographic section.

Gender	Frequency	Percentage
Women	48	68.6
Men	22	31.4
**Course of study**		
Bachelor’s degree	35	50
Master’s degree	6	8.6
Single-cycle Master’s degree	25	35.7
Doctorate/Master’s/Specialisation	4	5.7
**Department**		
Agriculture	3	4.3
Architecture	3	4.3
Biomedical Sciences	7	10
Chemical Sciences	8	11.4
Economic Sciences	6	8.6
History	6	8.6
Humanities and Social Sciences	12	17.1
Law	16	22.9
Medicine, Surgery and Pharmacy	4	5.7
Veterinary Medicine	5	7.1
**On-campus/off-campus student**		
Yes	43	61.4
No	27	38.6
**Status**		
Full-time	69	98.6
Part-time	1	1.4
**Nationality**		
Italian	65	92.9
Other country	5	7.1
**Work**		
No	57	81.4
Yes	13	18.6

**Table 2:** General reason for accessing counselling services.

	Frequency	Percentage
Effectively managing my emotions	36	51.4
Increase my psychosocial and academic resources, which will also be useful in continuing or completing my studies.	17	24.3
Address issues that affect my academic career	7	10
Improve study methods	4	5.6
Improving integration and adaptation to a new educational and social environment	3	4.3
Choosing a course of study at the end of current studies	3	4.3

**Table 3:** Paired sample *t*-test: differences in academic satisfaction (C-Sat) means.

Factors	M Pre-Test	M Post-Test	DS Pre-Test	DS Post-Test	t	*p*	Cohen’s d	Holm–Bonf. Sig.
Choice	15.39	16.82	3.64	3.50	−3.25	0.002	0.40	✓
Services	12.38	13.05	3.02	3.38	−1.89	0.062	0.20	✓
Relations	11.31	13.76	4.83	4.75	−4.00	<0.001	0.51	✓
Study	12.17	14.97	3.65	3.91	−5.47	<0.001	0.76	✓
Utility	15.47	16.53	3.69	4.33	−2.37	0.021	0.26	✗

Note. ✓ = significant after Holm–Bonferroni correction; ✗ = not significant after correction.

**Table 4:** Paired sample *t*-test: differences in Flourishing and SWLS means.

Factors	M Pre-Test	M Post-Test	DS Pre-Test	DS Post-Test	t	*p*	Cohen’s d	Holm–Bonf. Sig.
Flourishing	40.30	44.40	7.25	8.04	−3.95	0.001	0.53	✓
Life satisfaction	18.80	22.80	5.93	6.19	−5.18	<0.001	0.65	✓

Note. ✓ = significant after Holm–Bonferroni correction.

**Table 5:** Paired sample *t*-test: differences in “non-intellective” competences related to performance and academic satisfaction (C-Comp scale) means.

Factors	M Pre-Test	M Post-Test	DS Pre-Test	DS Post-Test	t	*p*	Cohen’s d	Holm–Bonf. Sig.
Family relationships	14.74	15.41	4.71	4.43	−1.57	0.120	0.14	✓
Student relationships	12.07	14.36	5.01	4.48	−3.94	<0.001	0.48	✓
Professors relationships	12.68	14.88	3.21	3.89	−4.65	<0.001	0.61	✓
Intrinsic motivation	13.55	14.21	3.34	3.84	−1.69	0.094	0.18	✓
Extrinsic motivation	12.05	12.76	4.52	4.23	−1.52	0.131	0.16	✓
Reaction to failure	10.43	12.71	4.53	4.22	−4.23	<0.001	0.52	✓
Learning assessment	13.31	15.15	3.22	3.20	−4.53	<0.001	0.57	✓
Time management	11.68	13.71	3.73	4.37	−3.95	<0.001	0.49	✓
Academic self-esteem	12.94	14.98	3.29	3.22	−4.99	<0.001	0.62	✓
Academic self-efficacy	14.84	16.49	3.67	2.95	−3.86	<0.001	0.49	✓
Dedication to study	14.32	15.32	4.00	3.88	−2.35	0.021	0.36	✗
Emotional control	8.41	10.85	4.07	4.07	−5.64	<0.001	0.59	✓

Note. ✓ = significant after Holm–Bonferroni correction; ✗ = not significant after correction.

**Table 6:** Paired sample *t*-test: differences in positive resources means.

Factors	M Pre-Test	M Post-Test	DS Pre-Test	DS Post-Test	t	*p*	Cohen’s d	Holm–Bonf. Sig.
Courage	26.08	30.57	7.21	7.36	−5.34	<0.001	0.61	✓
Optimism	14.65	17.05	4.60	5.02	−3.96	<0.001	0.49	✓
Hope	23.64	27.29	6.29	5.64	−4.70	<0.001	0.61	✓
Future orientation	52.72	55.04	9.85	11.19	−1.65	0.102	0.22	✓
Resilience	35.87	40.90	7.43	8.50	−4.54	<0.001	0.63	✓

Note. ✓ = significant after Holm–Bonferroni correction.

**Table 7:** Paired sample *t*-test: differences in satisfaction with performance and importance university means.

Factors	M Pre-Test	M Post-Test	DS Pre-Test	DS Post-Test	t	*p*	Cohen’s d	Holm–Bonf. Sig.
Satisfaction with performance	3.00	3.57	0.91	0.98	−4.46	<0.001	0.60	✓
Importance of university	4.27	4.14	0.63	0.66	1.63	0.106	0.20	✓

Note. ✓ = significant after Holm–Bonferroni correction.

**Table 8:** Changes in university life and life in general due to the counselling programme.

Changes in University Life	Frequency	Percentage
Yes	56	80
No	14	20
*If yes, which ones?*		
Improvement in academic self-esteem	31	44.3
Better study methods	14	20
Better ability to manage anxiety before exams	12	17.1
Career decision-making	7	10
No response	6	8.6
**Changes in life**		
Yes	54	77.1
No	16	22.9
*If yes, which ones?*		
Personal growth	41	58.6
Better ability to manage anxiety	17	24.3
Start of psychotherapy	4	5.7
No response	8	11.4

**Table 9:** Service response to the motivation for accessing it.

	Frequency	Percentage
1 = not at all	2	2.9
2	2	2.9
3	2	2.9
4	4	5.7
5	4	5.7
6	3	4.3
7	15	21.4
8	20	28.6
9	6	8.6
10 = completely	12	17.1

## Data Availability

The data presented in this study are available on request from the corresponding author.

## Supplementary Materials

The following supporting information can be downloaded at https://www.mdpi.com/article/10.3390/bs16030410/s1, Figure S1: Scores in personal well-being and academic satisfaction; Figure S2: Scores in academic skills; Figure S3: Scores in psychosocial resources.
