# Supplementary material for: The Impact of a University Counselling and Psychological Support Service Focused on Positive Resources and Student Well-Being
Source: Behav Sci (Basel). 2026 Mar 11;16(3):410. doi: 10.3390/bs16030410 (PMC13023995; doi:10.3390/bs16030410)
Supplement: Supplementary file 1 [file behavsci-16-00410-s001.zip › behavsci-4123570-supplementary.pdf]

Supplementary Materials

Student Profile Example

Dear Student,

the questionnaire you filled out represents a self-report in which students provide a description of themselves regarding the variables resulting from the questionnaire. Therefore, the results you will read are not about the skills and abilities one possesses but rather one's own perception of them. For this reason, the results “take a picture” of a current state that, with proper care and attention, is always open to change and improvement.

The assessment covers three general macro areas: personal well-being and academic satisfaction; academic skills; and psychosocial resources.

The following are graphs that represent the above areas and the constructs assessed within them, along with the relative scores obtained from your answers. In this regard, keep in mind that scores between 0 and 39 indicate areas of possible improvement; values between 40 and 60 represent scores around the mean (the exact mean is the value 50); scores above 60 represent your current strengths.

FIGURE S1. Scores in personal well-being and academic satisfaction

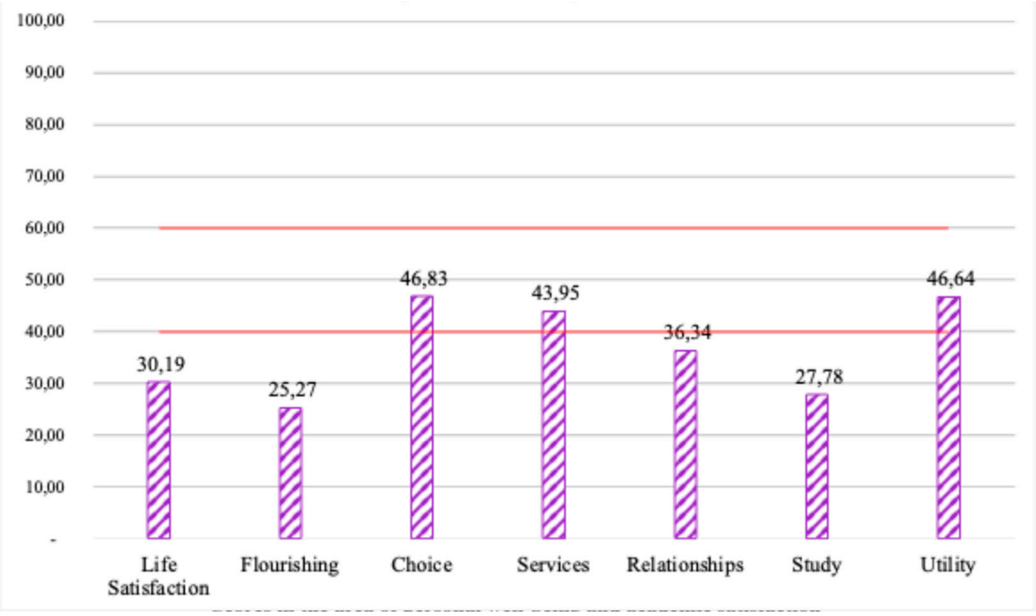

Life satisfaction represents the cognitive dimension of subjective well-being and is defined as a conscious qualitative evaluation of a person's life in relation to a set of personally established criteria. It is a dimension greatly influenced by both our mood of the moment and our personality traits.

Flourishing is a basic component of psychological well-being assessment and consists of the level of individual well-being you feel when you are achieving your fullest potential, abilities, skills and talents.

Academic satisfaction, in particular:

- **satisfaction with choice** indicates how satisfied you are with the course of study you have chosen
- **satisfaction with services** indicates how satisfied you are with the services offered by your university/academy/conservatory (classrooms, instruments, employees...)
- **satisfaction with relationships** indicates how satisfied you are with the relationships developed with your fellow students and professors

- **satisfaction with study** stands for how satisfied you are with your study method and results
- **satisfaction with utility** refers to how satisfied you are with your course of study relating to how useful it will be for joining the occupational market.

FIGURE S2. Scores in academic skills

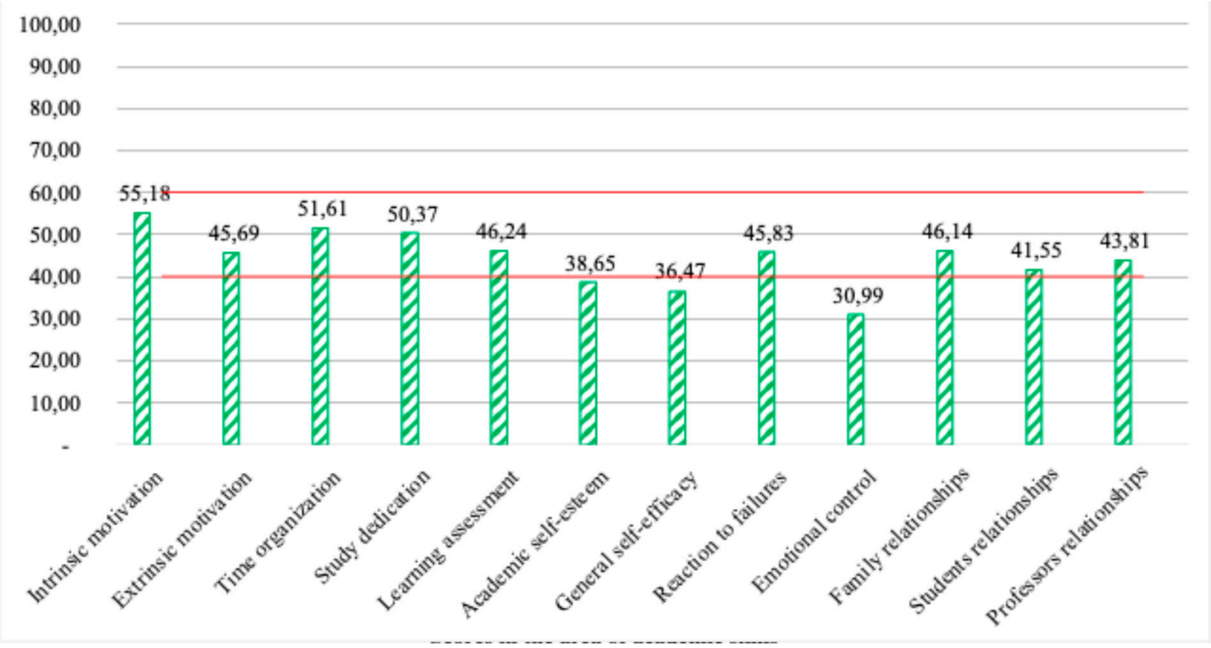

Intrinsic Motivation indicates the motivation and ability to self-motivate into studying and reaching academic achievements and goals for the pleasure of doing so and for the passion that leads us.

Extrinsic Motivation indicates the tendency to self-motivate to study and to achieve goals for the purpose of gaining external recognition (e.g., from family members or the social environment).

Time Organization indicates the ability to organize personal commitments, time and activities in such a way that the necessary space is devoted to study.

Dedication to study indicates the tendency to study with commitment and steadiness.

Learning assessment indicates the ability to evaluate the level of learning, for example, estimating how ready you are to take an exam.

Academic self-esteem represents the global assessment people make of themselves regarding their personal worth. In the academic context, it indicates the self-value as a student.

General self-efficacy indicates the subjective perception of being competent in coping with study and represents the belief or the confidence to achieve a specific academic goal or obtain a specific result on a specific academic task.

Reaction to failures indicates the ability to not become demoralized in response to study-related difficulties and the attempt to overcome the problems encountered.

Emotional control stands for the ability to effectively manage personal emotions, such as those related to facing university exams.

Students relationships indicate a tendency to share difficulties with peers and seek their help.

Family relationships indicate the tendency to share with family members the experiences and emotions related to personal journey through the course of study.

Professors relationships indicate a tendency to build a good relationship with professors and seek their help to improve learning.

FIGURE S3. Scores in psychosocial resources

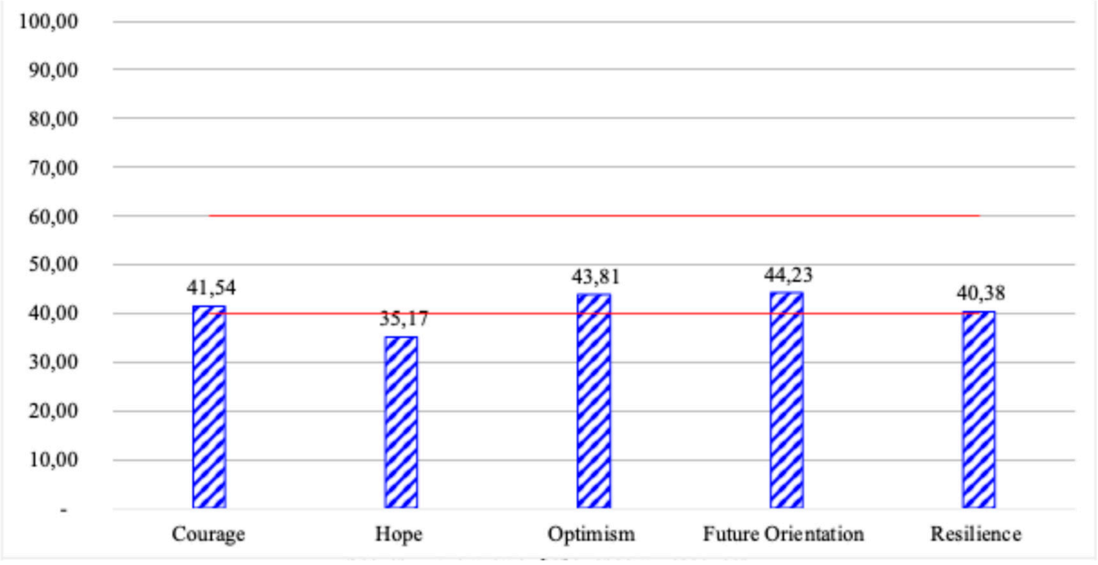

Courage indicates the propensity to make efforts and to persist over time toward a goal despite a subjective perception of fear.

Hope represents the ability to set goals, identify the strategies needed to achieve them, and motivation toward the possibility of reaching specific outcomes.

Optimism indicates the propensity to believe that positive future life scenarios can be built and achieved, assigning primarily to the person's own ability to do so (and not, for example, to luck or fate).

Future orientation indicates the tendency to focus on the future by planning behaviors and by being careful about health, facing the path with energy and control of actions.

Resilience represents the ability to persist to life's difficulties even in the presence of particularly negative events.
